# Supplementary material for: The Use of Post-Natal Skeleton Development as Sensitive Preclinical Model to Test the Quality of Alternative Protein Sources in the Diet
Source: Nutrients. 2022 Sep 13;14(18):3769. doi: 10.3390/nu14183769 (PMC9501083; doi:10.3390/nu14183769)
Supplement: Supplementary file 1 [file nutrients-14-03769-s001.zip › nutrients-1876751-supplementary.pdf]

**Table S1. Statistically analyses of body weight and length.** Statistical differences of body weight and length presented in graphs 1E and 1F according to measurements days. Different superscript letters are significantly different ($P < 0.05$) by one-way ANOVA followed by Tukey's test.

| Day | PD-Ctrl | PD-Soy | PD-Spl | PD-CP/I | PD-CP/F | PD-Fly |
|---|---|---|---|---|---|---|
| | | | **Body Weight** | | | |
| 1 | a | a | a | a | a | a |
| 4 | a | b | b | b | ab | ab |
| 8 | a | c | c | c | bc | ab |
| 11 | a | d | cd | d | bc | b |
| 15-39 | a | c | c | c | b | b |
| 43 | a | d | d | d | c | b |
| | | | **Body Length** | | | |
| 1 | a | a | a | a | a | a |
| 8 | a | b | ab | ab | ab | ab |
| 15 | a | c | bc | bc | ab | ab |
| 22 | a | c | bc | bc | b | b |
| 29-43 | a | c | c | c | b | b |

**Table S2. Comparison between PD-Ctrl diet's essential amino acids content and the requirements for growing rats.** Values are expressed as g/kg diet. According to NRC's amino acids requirements for growing rats.

| g/kg diet | PD-Ctrl diet | Growing Rats Requirements |
|---|---|---|
| **Histidine** | 1.9 | 2.8 |
| **Isoleucine** | 3.0 | 6.2 |
| **Leucine** | 6.5 | 10.7 |
| **Lysine** | 6.8 | 9.2 |
| **Methionine + Cysteine** | 3.7 | 9.8 |
| **Phenylalanine + Tyrosine** | 7.7 | 10.2 |
| **Threonine** | 3.1 | 6.2 |
| **Valine** | 4.2 | 7.4 |
| **Tryptophan** | 0.6 | 2 |

**Table S3. Essential amino acids profile of the tested protein sources compared to humans requirements.** Values are expressed as g/100g crude protein. Limiting amino acids are underlined. According to WHO's amino acid requirements for 3-10 years old children.

| g/100g crude protein | Caseinate | Soy Isolate | Spirulina Powder | Chickpea Isolate | Chickpea Flour | Fly Larva Protein | Humans Requirements |
|---|---|---|---|---|---|---|---|
| **Histidine** | 2.3 | 2.1 | 1.6 | 2.3 | 4.2 | 2.3 | **1.6** |
| **Isoleucine** | 3.8 | 3.2 | 4.3 | 3.8 | 4.0 | 4.1 | **3.1** |
| **Leucine** | 8.1 | 6.6 | 7.0 | 7.4 | 8.3 | 7.2 | **6.1** |
| **Lysine** | 8.4 | 6.6 | <u>4.7</u> | 7.3 | 9.8 | 7.1 | **4.8** |
| **Methionine + Cysteine** | 3.1 | <u>1.9</u> | 3.0 | <u>2.1</u> | 3.2 | 2.5 | **2.4** |
| **Phenylalanine + Tyrosine** | 9.5 | 7.6 | 7.5 | 8.7 | 16.4 | 10.6 | **4.1** |
| **Threonine** | 3.9 | 3.4 | 4.4 | 3.2 | 4.4 | 4.3 | **2.5** |
| **Valine** | 5.2 | <u>3.3</u> | 4.1 | 4.0 | 4.4 | 4.5 | **4** |
| **Tryptophan** | 0.8 | 0.7 | 1.0 | <u>0.6</u> | <u>0.5</u> | 1.0 | **0.66** |
